# Lab Protocol Paper: Use of a High-throughput, Multiplex Reverse-transcription Quantitative Polymerase Chain Reaction Assay for Detection of Sabin Oral Polio Vaccine in Fecal Samples

**DOI:** 10.1093/cid/ciy648

**Published:** 2018-10-30

**Authors:** Christopher van Hoorebeke, ChunHong Huang, Sean Leary, Marisa Holubar, Jonathan Altamirano, Meira S Halpern, Marvin Sommer, Yvonne Maldonado

**Affiliations:** Stanford University School of Medicine, California

**Keywords:** Oral polio vaccine (OPV), Mexico, polio, RT-qPCR, detection

## Abstract

**Background:**

Global polio eradication efforts rely in part on molecular methods of detecting polioviruses, both wild and vaccine strains, from human and environmental samples. Previous assays used for detection of Sabin oral polio vaccine (OPV) in fecal samples have been labor and time intensive and vary in their sensitivity and specificity.

**Methods:**

We developed a high-throughput, multiplex reverse-transcription quantitative polymerase chain reaction assay able to detect all 3 OPV strains in fecal samples. The assay used a KingFisher Duo Prime system for viral RNA isolation and extraction. Positive samples were retested and Sanger sequenced for verification of Sabin serotype identity.

**Results:**

The 95% lower limit of detection was determined to be 3 copies per reaction for Sabin 1 and 3 and 4 copies per reaction for Sabin 2, with no cross-reactivity between the 3 serotypes and their primers. A total of 554 samples (3.6%) were positive, with 304 positive samples (54.9%) containing >1 serotype. Of the positive samples, 476 (85.9%) contained enough RNA to be sequenced, and of these all sequences were Sabin serotypes. The previous assay we used could process 48 samples in a 10-hour period, whereas the new assay processed >100 samples in 6 hours.

**Conclusions:**

The new high-throughput, multiplex reverse-transcription quantitative polymerase chain reaction assay allowed for sensitive and specific detection of OPV serotypes while greatly decreasing sample handling and processing time. We were able to sequence 72.4% of the 210 positive samples in the cycle threshold range of 35–37.

As part of the Global Polio Eradication Initiative, it is critical to terminate the global use of live, attenuated oral polio vaccine (OPV). OPV, which usually included 3 serotypes of attenuated, live polioviruses, was used primarily in developing countries owing to its lower cost, ease of administration, and increased intestinal immunity compared with inactivated polio vaccine (IPV), which is parenterally administered [1]. A major drawback of OPV use is that it can cause paralytic poliomyelitis through 2 distinct mechanisms. OPV neurorevertant mutants are known to cause vaccine-associated paralytic poliomyelitis (VAPP), estimated to occur in 1 per 750000 primary OPV doses [2]. The second type of neurovirulent revertant mutations is associated with long-term circulation of vaccine-derived polio viruses (VDPVs), defined as polioviruses with VP1 region genomes that include ≥6 nucleotide changes from the parental Sabin strains for type 2 and ≥10 nucleotide changes for types 1 and 3, detected using sequencing [3]. In contrast, because IPV is composed of killed poliovirus, there is no potential for either VAPP or VDPV neurovirulent revertant mutations. Neurorevertant mutations are distinct for VDPV and VAPP. VDPV mutations occur randomly and accumulate over time as OPV viruses circulate. By contrast, VAPP occurs as a result of point mutations in stem-loop V of the 5’ untranslated region (UTR) [4]. Neurovirulent revertant mutations occur at residue 480 (G to A) in OPV serotype 1 (OPV-1), 481 (A to G) in OPV serotype 2 (OPV-2), and 472 (U to C) in OPV serotype 3 (OPV-3) [4, 5]. These residues correspond to an internal ribosomal entry site for proteins involved in the translation and replication of viral RNA, and they are used as attenuation markers owing to their ability to inhibit translation [4].

Between January 2006 and May 2016, >94% of circulating VDPVs detected were serotype 2, whereas no wild-type poliovirus 2 has been detected since 1999 [6]. Because of these findings, in April 2016 a shift in global immunization strategy included replacing the manufacturing and administration of trivalent OPV with the administration of bivalent OPV, containing only serotypes 1 and 3, complemented with a dose of IPV [6]. Wild-type poliovirus 3 has not been detected since November 2012, whereas wild-type poliovirus 1 has still been recently detected, with only 22 paralytic cases identified in 2017 [7]. It will be of critical importance to continue IPV administration until no more VDPVs are detected globally, and the World Health Organization has recommended that all countries administer ≥2 doses of IPV during routine immunization for ≥10 years after polio eradication is certified [8]. Finally, because of the low risk of paralytic polio cases, the probability of asymptomatic polio infection and shedding, and the infeasibility of conducting surveillance via testing of stool samples, environmental sampling will be of great importance with regard to global identification of VDPVs.

Rural villages in Mexico provide ideal settings for analyzing fecal shedding and transmission of OPV. Since 2007, the country has provided routine polio immunization with IPV and has 2 national immunization weeks per year when all children <5 years of age are offered vaccination with OPV. Thus, dual administration of both IPV and OPV in this environment provides an opportunity to study factors that affect shedding and transmission of OPV during and after national immunization weeks, as well as the potential impact of IPV. In a current study, we identified 3 indigenous Mexican villages where we studied the shedding and household and community transmission of OPV [9–10]. In the current study, a total of approximately 15000 fecal samples were collected over a 10-week study period after the initial study national immunization week.

It is common laboratory practice to isolate poliovirus from OPV vaccinees through the treatment of stool samples with chloroform and inoculation in different cell cultures with the stool supernatant. To identify serotype, a second passage in cell culture is generally required, with further analysis by polymerase chain reaction (PCR) [8]. Cell culture, when compared with nucleic acid extraction coupled with reverse-transcription (RT) quantitative PCR (qPCR), is time, labor, and resource intensive, taking weeks to process a sample to completion. In addition, cell culture methods have been found to be approximately 100-fold less sensitive than qPCR [11]. Owing to the large scale of the project. our laboratory adopted and modified a cell culture–free, high-throughput, multiplex assay that could detect all 3 OPV strains in fecal samples.

## METHODS AND MATERIALS

### Study Design and Population

The study protocol was reviewed and approved by (1) Stanford University’s Institutional Review Board, (2) the Ethics, Biosafety, and Research Committees of the Mexican National Institute of Public Health, (3) the Mexican National Center for Infant and Adolescent Health, (4) the Mexican Center for Infectious Disease Research, (5) the Veracruz Institute of Research in Health, and (6) and the Public Health Center of Orizaba, Veracruz, Mexico.

Each village received a different amount of OPV coverage as part of the study: 70% of households in Capoluca, 30% in Campo Grande, and 10% in Tuxpanguillo. In each village, recruited families were randomized to receive of OPV based on these percentages, resulting in 155 vaccinated households across the 3 localities. After enrollment, 10 stool samples were scheduled for collection from each participant, 1 baseline sample collected before vaccination, and 9 samples collected 1, 4, 7, 10, 14, 21, 28, 51, and 71 days after vaccination. During each visit, health information, travel and visit details, and records for vaccinations received by any children <5 years old during the study period, were collected via follow-up surveys. (Details are provided elsewhere [12]).

### Sample Processing

Stool samples were collected using containers with screw caps for adults and children able to use the toilet. For children who used diapers, parents were asked to keep the diapers for the field team, who collected samples from the diapers. Participants were asked to avoid mixing their sample with urine, when possible. Samples were transported and registered at community health centers on the day of collection, where samples were divided into 1-mL vials. Unique identification numbers were recorded on each vial via barcode, and these vials were then stored in cryoboxes with 81 numbered slots. Samples were then preserved at −70°C at facilities in Orizaba, Veracruz, Mexico. Quality control checks and inventory of samples were performed daily. Samples were shipped in batches on dry ice to the Stanford laboratory, where they were maintained at −80°C until tested.

### RNA Extraction and Isolation

Frozen stool samples were thawed on ice, and a 10% stool suspension was created in 500 μL of phosphate-buffered saline. The stool suspension were lysed using a MagNA Lyser (Roche) at 6500 rpm for two 30-second periods, with 30 seconds on a cooling block (4°C) in between to keep samples from degrading because of excessive heat. The lysed suspensions were centrifuged at 8000*g* for 2 minutes to pellet any debris. Next, 200 μL of the supernatant was added to a 96–deep well plate for processing in a KingFisher Duo Prime system (Thermo Scientific). Each 200-μL aliquot was spiked with 1 μL of the bacteriophage MS2 (American Type Culture Collection), to act as an internal positive control for RNA extraction. If the MS2 cycle threshold (Ct) reading during the RT-qPCR assay was >37, the run was considered invalid and the sample was reprocessed. Carrier RNA (2 μL) (Ambion; Life Technologies) was added to enhance the nucleic acid yield. Viral RNA was extracted from the supernatant according to the manufacturer’s instructions (Invitrogen or Thermo Fisher Scientific) for isolating viral RNA. The viral RNA was eluted into 50 μL of elution buffer (Invitrogen) and stored at −20°C until it was ready for RT-PCR processing.

### RT-qPCR Assays

A single RT-qPCR cycle was performed by adding 5 μL of extracted viral RNA with a mix containing 1 μL of 10 mmol/L deoxyribonucleotide triphosphates (final concentration, 500 μmol/L), 6 μL of sterile water, and 1 μL of random hexamer primer, for an initial reaction volume of 13 μL. Serotype-specific cell culture stocks obtained from the Centers for Disease Control and Prevention were used as positive controls, containing 2 μL of OPV, 3 μL of sterile water, and 8 μL of the above mix. A negative control was included as well. This reaction was heated to 65°C for 5 minutes and then cooled on ice (for approximately 15 minutes) to allow for annealing of primers. Next, an enzyme mix containing 4 μL of 5× First-Strand Buffer, 1 μL of 0.1 mol/L dithiothreitol, 1 μL of RNaseOUT Recombinant Ribonuclease Inhibitor (40 U/μL), and 1 μL of SuperScript lll Reverse Transcriptase (200 U) was added to the previous reaction, resulting in a final reaction volume of 20 μL (all reagents in this mix were obtained from Invitrogen). Samples were vortexed and centrifuged after each addition step to ensure proper mixing. The reactions were performed in a 96-well thermal cycler (Applied Biosystems Veriti), using the following cycling parameters: 5 minutes at 25°C, 60 minutes at 55°C, and 15 minutes at 75°C. The resulting complementary DNA (cDNA) was then held at 4°C for 10 minutes, 45 μL of sterile water was added to each sample, and the samples were either kept on a cooling block or stored at −20°C until ready to be processed by qPCR.

### Primer and Probe Design

The probes and primers (Table 1) were adopted from Kilpatrick et al [13] and the Centers for Disease Control and Prevention’s poliovirus diagnostic PCR [14], with slight modifications to the fluorophores and quenchers of the probes. The primers correspond to 95-, 70-, and 54-nucleotide portions of the highly, conserved *VP1* gene for OPV-1, OPV-2, and OPV-3 respectively. Our laboratory adapted the probes from Kilpatrick et al [13], as following: the probe used in the detection of serotype 1 used Black-Hole Quencher 2, the probe for serotype 2 used Zen as an internal quencher 9 nucleotides downstream of the fluorophore FAM and used the quencher Iowa Black FQ, and the serotype 3 probe contained the fluorophore Texas Red instead of ROX [14]. For qPCR analysis a composite primer (Table 2) containing the sequence of all 3 Sabin OPV serotype primers, found in Table 1, was used. All probes and primers were obtained from IDT DNA.

**Table 1.** Primer and Probes for Quantitative Polymerase Chain Reaction and Sequencing

Name	Sequence (5’ → 3’)	Nucleotide Location	Concentration, nmol/L
Serotype1			
Forward	AGGTCAGATGCTTGAAAGC	2505–2523	300
Reverse	CCACTGGCTTCAGTGTTT	2600–2583	300
Probe	Cy5-TTGCCGCCCCCACCGTTTCACGGA-BHQ2	2540–2563	125
Serotype 2			
Forward	CCGTTGAAGGGATTACTAAA	2525–2544	300
Reverse	CGGCTTTGTGTCAGGCA	2595–2579	300
Probe	6FAM-ATTGGTTCC-ZEN-CCCGACTTCCACCAAT-IBFQ	2550–2572	150
Serotype3			
Forward	AGGGCGCCCTAACTTT	2537–2552	400
Reverse	TTAGTATCAGGTAAGCTATC	2591–2572	400
Probe	TexasRed-X-TCACTCCCGAAGCAACAG-BHQ2	2554–2571	300
T7Y7 forward^a^	*GCGGCCGCTAATACGACTCACT ATAGG*GGGTTTGTGTCAGCCTGTAATGA	2419–2441	400
Q8R reverse^a^	*TACGGTAGCAGAGACTTGGT CT*AAGAGGTCTCTRTTCCACAT	3527–3508	400

Abbreviations: 6FAM, 6-carboxyfluorescein; BHQ2, Black Hole Quencher-2; IBFQ, Iowa Black Fluorescent Quencher; TexasRed-X, Texas Red-X NHS ester.

^a^Nucleotides in italics are modified 5’ sequence tags. R can be either adenine or guanine.

**Table 2.** Synthetic Double-stranded DNA Oligonucleotides Encoding Sabin Sequences

Target	Sequence (5’ → 3’)
*VP1*	
Composite serotypes 1, 2, and 3	CACAGGGGTTAGGTCAGATGCTTGAAA GCATGATTGACAACACAGTCCGTGAAACG GTGGGGGCGGCAACGTCTAGAGACGCTC TCCCAAACACTGAAGCCAGTGGACCAGCA CACTGAGGGGGCCGTTGAAGGGATTACTAAA AATGCATTGGTTCCCCCGACTTCCACCAATAGCC TGCCTGACACAAAGCCGAGCGGTCCAGCGAAG TTGCACAGGGCGCCCTAACTTTGTCACTCCCGAA GCAACAGGATAGCTTACCTGATACTAAGGCCAGTGGC

### Calculating Lower Limits of Detection

The 95% lower limit of detection (LLOD) for each serotype in the multiplex assay was determined. The LLOD was found through probit analysis ,using RNA extracted from tissue culture controls (Hep2 cells). A synthetic composite oligonucleotide standard (IDT DNA) containing 10^9^ viral RNA copies of Sabin OPV-1–3 was used to create a 9-fold 1:10 serial dilution, ranging from 1 to 10^8^ copies per reaction (Table 2). To determine the LLOD of the assay, the Ct values from culture stock dilutions were compared with those of the composite standard. Sabin OPV-1 and OPV-2 were tested 7 times, and OPV-3 was tested 6 times, all in triplicate. Control stocks were used to compare different primer/probe sets.

### Real-time PCR

A 20-μL reaction of 5-μL cDNA and 15-μL IQ Multiplex Powermix containing the primers and probes for each sample was run in triplicate on a 384-well plate (see Table 1 for concentrations). Positive control stocks from cell culture, 9 serial dilution (1:10) standards (IDT DNA), and 2 nontemplate controls were included for each run. The CFX384 Real-Time System thermocycler was used under the following conditions: 52°C for 2 minutes, and 95°C for 10 minutes, followed by 40 cycles of 95°C for 15 seconds and 60°C for 1 minute. The baseline threshold of 300 relative fluorescence units was used to determine the Ct for each reaction. A Ct <37 was regarded as positive and samples with ≥2 of 3 reactions below the threshold of 37 were considered positive and set aside for further analysis. The Multiplex Powermix, 384-well plates, and thermocycler were sourced from Bio-Rad.

### Sequencing of Positive Results

First, 5 μL of isolated RNA was reverse-transcribed for 5 hours using SuperScript III reverse-transcriptase (Thermo Fisher Scientific) or for 1 hour using the PrimeScript RT Reagent Kit (TaKaRa Bio), according to the manufacturer’s instructions, using primer Q8R modified with a 5’ sequence tag. The approximately 900 nucleotides encoding the viral structural proteins VP1-2A junction region were amplified using the modified primer Q8R and primer T7Y7, also modified with a 5’ sequence tag (see Table 1 for sequences and genomic location) [15]. Each 50-μL reaction contained LongAmp Hot Start Taq 2× Master mix (New England BioLabs), each primer at 400 nmol/L, 400 ng/μL bovine serum albumin, and 2 μL of cDNA.

The reactions were performed in an Applied Biosystems Veriti 96-well thermal cycler, using the following cycling parameters: 94°C for 2 minutes; 40 cycles of 94°C for 30 seconds, 70°C for 1 second (ramp rate, 20%), 55°C for 45 seconds (ramp rate, 20%), and 65°C for 1 minute 20 seconds, with final extension for 10 minutes at 65°C. The PCR products were electrophoresed on a 1.5% agarose gel and visualized by means of ethidium bromide staining using a Gel Doc Molecular Imager (Bio-Rad). The approximately 900-nucleotide amplicon was excised and purified using the QIAquick Gel Extraction Kit (Qiagen). The purified PCR products had measured absorbance at 260 and 280 nm, using a Synergy H1 Hybrid reader (BioTek). The band was then sent to QuintaraBio for sequencing.

## RESULTS

The assay developed by our laboratory had a 95% LLOD for each serotype as follows: 3 copies per reaction for OPV-1 and OPV-3, and 4 copies per reaction for OPV-2. There was an inverse linear relationship in the stool sampling assay between the amount of virus and the Ct value, such that lower Ct values corresponded to higher concentration of virus. There was no cross reactivity between the different serotypes and primers. No nonpolio enteroviruses (NPEVs) were detected in the sequenced positive samples.

After initial processing, 722 (4.7%) of the samples were labeled as negative owing to failed MS2 controls. All samples with no MS2 control readings were retested up to 2 times to rule out poor sample handling. After retesting these samples, 178 (24.7%) remained unresolved as a result of failed MS2 controls, and of the 722 retested samples, Sabin OPV was detected in only 1 (0.14%).

Of the 15 218 samples analyzed, 554 (3.6%) were positive for ≥1 Sabin OPV serotype, with OPV-2 being the most prevalent, found in 405 (73.1%) of the positive samples. OPV-1 and OPV-3 were found in 268 (48.4%) and 318 (57.4%) of the samples, respectively. More than half of the positive samples contained >1 serotype with 171 (30.9%), and 133 (24.0%) of the Sabin OPV–positive samples contained 2 and 3 serotypes, respectively. If a sample was positive for 2 serotypes, Sabin OPV-2 and OPV-3 were the most common pair. Shedding trends for the 3 serotypes varied over time. Sabin OPV-1 shedding remained at constant low levels throughout the study, OPV-2 shedding was higher toward the beginning (days 1–10), and OPV-3 was higher at the end (days 14–51).

Of the 554 positive samples, 476 (85.9%) contained enough cDNA to be sequenced, and a dominate serotype was recorded in 307 (64.5%). Of the sequenced samples, the dominant serotype could not be differentiated in 169 (35.5%), and these were labeled “mixed peaks”; Sabin OPV-1, OPV-2, and OPV-3 accounted for 47 (15.3%), t115 (37.5%), and 145 (47.2%) of the recorded “dominant peaks.”

## DISCUSSION

Previous assays used by our laboratory and others targeted the 5’ UTR of the poliovirus genome. The 5’ UTR is a region of interest because it contains markers for attenuation, particularly in stem-loop V, which allows for the tracking of VAPP and potential VDPVs. Targeting the 5’ UTR was found to be problematic, because the sequence is highly conserved throughout members of the enterovirus group. Therefore, depending on the time of year of sampling, there could be a significant proportion of false-positive results from NPEVs. In an earlier study, through careful genomic analysis, Kilpatrick et al [15] designed primers targeting the VP1 region. A PCR assay using these primers was able to detect and identify approximately 250–2500 molecules per reaction for Sabin 1 and 2 and 2500–25 000 molecules per reaction for the smaller Sabin 3 amplification product [15]. Our laboratory adapted a multiplex assay developed by Kilpatrick et al [13], which used the VP1 primers and probes. The probes and quenchers from that study were modified to increase the sensitivity for Sabin OPV-1, OPV-2, and OPV-3: 80, 60, and 800 fold, respectively. Previous assays generally used a Ct cutoff of 30–35. By increasing the sensitivity of the assay, our laboratory was able to use 37 as the Ct cutoff for a positive sample. A total of 210 positive samples were in the range of 35–37, of which we were able to sequence 152 (72.4%).

The benefits and advantages of RT-qPCR over cell culture methods have been documented extensively [11]. Coupled with the development of multiplex assaying, PCR has dramatically decreased sample processing time, allowing for the efficient detection of serotype specific polioviruses from stool samples. By using the KingFisher Duo Prime system and MagMAX Viral RNA Isolation Kit to isolate and extract viral RNA from stool samples, our laboratory was able to decrease processing time and the amount of labor required. To rule out improper laboratory technique, we analyzed samples in which the MS2 internal control initially failed up to a total of 3 times. The viral RNA extraction protocol was highly efficient, with only 1.2% potential false-negatives. According to Shulman et al [16], up to 7.6% of stool samples processed with the KingFisher Duo Prime system may have RT-qPCR inhibitors present. These inhibitors may be endogenous, such as metabolites from drugs or prescriptions, or exogenous, including chelating agents found in PCR reagents. When optimized, our laboratory’s previous assay could process 48 samples per 384-well plate over a span of ≥10 hours. The current assay could process 108 samples per 384-well plate in 6 hours, more than doubling the amount of samples processed in almost half the time.

Owing to the nature of viruses and their rapid evolution, it was important for our laboratory to sequence the positive results obtained in order to rule out the possibility of false positives as a result of NPEVs. By using mixed-base primers published by Kilpatrick et al [13], we were able to sequence a majority of the positive samples. No NPEVs were sequenced from the positive samples, a consequence of the highly specific primers used. Further analysis through whole-genome sequencing/deep sequencing would provide greater insight into the varying prevalence of the serotypes in circulation, as well as the progress of mutations, including potential neurovirulent revertant mutations, as Troy et al [17] published for a previous environmental sampling study in Mexico.

The current literature shows a varied range for the proportion of positive samples collected from populations challenged with OPV [18]. In our current study, approximately 3.5% of the samples from OPV vaccinated population were positive for Sabin OPV. After taking into account that 10%, 30%, and 70% of the population were vaccinated, our results compare favorably with those in the literature. Sabin OPV was detected throughout the 71-day sample collection period. The length of shedding is consistent with that in a previous Mexico field study [19]. In-depth epidemiological analysis by our laboratory’s statisticians is published elsewhere [9–10].

In summary, we have developed a high-throughput assay for the detection of Sabin OPV by optimizing a nucleic acid isolation protocol as well as adopting and adapting a previously published multiplex Q-PCR assay. As the Global Polio Eradication Initiative nears the final stages, poliovirus detection through environmental sampling will become more important. It will be critical to have protocols in place to allow for the highly specific, sensitive, and rapid detection of poliovirus from large numbers of clinical and environmental samples. Future work done by our laboratory includes transitioning this assay from stool to sewage sampling, as environmental samples were collected throughout and after our field study in Mexico. Analysis of sewage samples, combined with the data from this study, will provide further details into the persistence of Sabin OPV in remote, rural communities. This will allow for the logical and rational development of vaccination policy as we move closer to the overall global goals of polio eradication and polio vaccine cessation.

## Notes

***Acknowledgments.*** We thank the families who participated in this study, the large field teams in Mexico who performed data and sample collection, and the broader team at Stanford University, including Aisha Talib, Jennifer Lee Kang, and Rasika Behl.

***Financial support*.** This work was supported by The Bill & Melinda Gates Foundation (grant OPP1116910).

***Supplement sponsorship.*** **This article appears as part of the supplement “Polio endgame and beyond: Vaccine choices, transmission dynamics, and surveillance implications,” sponsored by The Bill & Melinda Gates Foundation.

***Potential conflicts of interest.*** Y. M. reports grants from The Bill & Melinda Gates Foundation, during the conduct of the study, and personal fees and other support from Pfizer, outside the submitted work. All other authors report no potential conflicts. All authors have submitted the ICMJE Form for Disclosure of Potential Conflicts of Interest. Conflicts that the editors consider relevant to the content of the manuscript have been disclosed.
